# Changes in Patch Features May Exacerbate or Compensate for the Effect of Habitat Loss on Forest Bird Populations

**DOI:** 10.1371/journal.pone.0021596

**Published:** 2011-06-28

**Authors:** Ainhoa Magrach, Asier R. Larrinaga, Luis Santamaría

**Affiliations:** Mediterranean Institute for Advanced Studies (IMEDEA, CSIC-UIB), Esporles, Mallorca, Balearic Islands, Spain; University of Zurich, Switzerland

## Abstract

One and a half centuries after Darwin visited Chiloe Island, what he described as “…an island covered by one great forest…” has lost two-thirds of its forested areas. At this biodiversity hotspot, forest surface is becoming increasingly fragmented due to unregulated logging, clearing for pastures and replacement by exotic tree plantations. Decrease in patch size, increased isolation and “edge effects” can influence the persistence of forest species in remnant fragments. We assessed how these variables affect local density for six forest birds, chosen to include the most important seed dispersers (four species) and bird pollinators (two species, one of which acts also as seed disperser), plus the most common insectivore (*Aphrastura spinicauda*). Based on cue-count point surveys (8 points per fragment), we estimated bird densities for each species in 22 forest fragments of varying size, shape, isolation and internal-habitat structure (e.g. tree size and epiphyte cover). Bird densities varied with fragment connectivity (three species) and shape (three species), but none of the species was significantly affected by patch size. Satellite image analyses revealed that, from 1985 to 2008, forested area decreased by 8.8% and the remaining forest fragments became 16% smaller, 58–73% more isolated and 11–50% more regular. During that period, bird density estimates for the northern part of Chiloé (covering an area of 1214.75 km^2^) decreased for one species (elaenia), increased for another two (chucao and hummingbird) and did not vary for three (rayadito, thrust and blackbird). For the first three species, changes in patch features respectively exacerbated, balanced and overcame the effects of forest loss on bird population size (landscape-level abundance). Hence, changes in patch features can modulate the effect of habitat fragmentation on forest birds, suggesting that spatial planning (guided by spatially-explicit models) can be an effective tool to facilitate their conservation.

## Introduction

Habitat fragmentation can be seen as a state of discontinuity in the spatial distribution of resources and conditions that affects occupancy, reproduction, or survival in a particular set of species [1]. But it can also be understood as the process by which this discontinuity arises, a process that has been described extensively as one of the major threats to biodiversity worldwide [2]–[6]. However, several authors have criticized this concept of fragmentation for its ambiguity [7] and because it provides an oversimplified explanation for too complex a process. Indeed, it is often used as if it were a unitary phenomenon [8] - neglecting the fact that it is diverse and comprises multiple components, where direct and indirect effects may have contradictory consequences for the different species inhabiting the fragmented landscape [9]. A recent review [10], suggests that the global process of habitat fragmentation should be decomposed into two different components: habitat loss and fragmentation *per se*. The latter is mediated by changes in the characteristics of the patches (e.g. patch size and isolation, the influence of edge ecotones, within-patch habitat quality) that are independent of habitat loss. Here, we will adhere to the use of fragmentation to refer to the process encompassing both habitat loss and fragmentation *per se*, while using the latter term when referring to the breaking apart of the habitat independent of habitat loss.

Two of the most studied effects of fragmentation are a reduction in patch area and an increase in patch isolation [10]; however, many studies fail to address them simultaneously (albeit see [11]). Further, studies that separate these components have nurtured an ongoing debate as to which of the two is the most important aspect: the total area of remaining habitat (or land cover elements) or the configuration of these remnants. Many authors claim total area to have a greater effect than configuration [10]. Nevertheless, recent studies have proved that this is not such a general trend and that it strongly depends on the needs of focal organisms [12]. First, responses to habitat area have been shown to range from positive to negative among different species [13]. Second, effects of isolation over species persistence are also highly variable [11]; patch isolation can be a very important predictor of species presence for organisms that are able to use the intervening matrix [14], and different species may differ in their abilities to use the fragmented habitat and the intervening matrix. For this reason, evaluations of the relative importance of habitat loss vs. configuration in fragmentation processes may require the simultaneous study of several species within a given guild or community.

Moreover, studies that address the relative effect of patch area and isolation have often failed to account for the simultaneous, often correlated effect of other variables - such as patch shape, penetration distances of edge effects or the internal characteristics of patches (although see [15]). Nevertheless, several recent reviews have acknowledged their increasing importance in human-altered landscapes (e.g. [9], [16]). Patch shape has been linked to the probability of species encountering edges in an area, which is maximized for convolutedly-shaped fragments in contrast with more compact ones [9]. Edge effects can extend over various distances towards the interior of the remaining fragments, depending on the focal organism [17], the type of edge [18] and the characteristics of the surrounding habitat [19]; thus requiring the evaluation of their penetration distance for the different study species [19]. Finally, the internal characteristics of patches are also likely to determine the abundance and, ultimately, the persistence of focal species within them.

To obtain a causal understanding of the changes in species distribution and abundance due to the fragmentation process, it is of key importance to separate the contribution of all of its associated components (e.g., changes in patch shape and isolation, edge effects) [7]. Since equivalent spatial patterns can be caused by totally different processes [20], the understanding of these relative contributions is a likely precondition for the development of effective conservation strategies. Such strategies should be aimed at minimizing the primary negative effects of fragmentation (e.g. habitat loss *versus* habitat isolation, [10]), specifically considering the spatial scale at which such threats operate [21].

This approach, however, needs to specifically consider the specificity of the effects caused by the different components of fragmentation. Responses to patch shape, edge effects and internal characteristics are likely to vary among different organisms, as has already been reported for patch size and connectivity. Indeed, not all species are affected negatively by the modification of the areas they inhabit; some species remain unaffected [19], [22] or even benefit from these conditions [19], [23]. The resulting variation stresses again the need for undertaking multi-species comparisons to address the relative contribution of the various processes that characterize habitat fragmentation.

**Figure 1:**
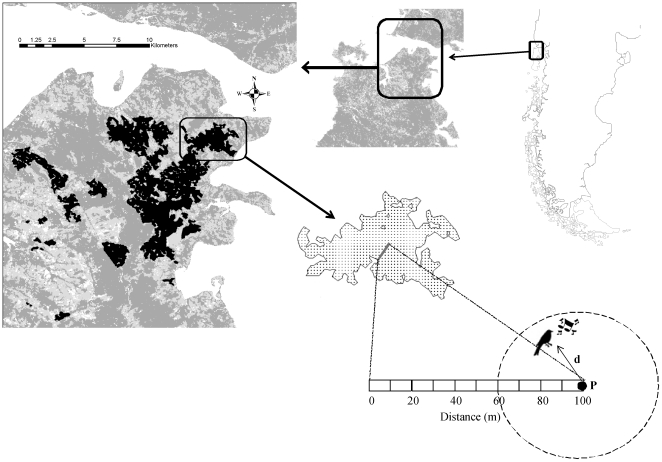
Study area in the northern part of Chiloe Island (southern Chile). Dark grey areas indicate forest fragments and patches selected for the study are inblack. Enlarged area shows one of the forest patches studied with an example of the two points sampled within each of the four transects (located, in this case, at 25 and 100 m from the nearest edge).

Our study addresses both the effects of the process of fragmentation, and the relative contributions of its different components, on the bird fauna of native, temperate austral forests. First we address the relative contribution of landscape configuration (connectivity), patch characteristics (size and shape) and patch internal features (tree size and epiphyte abundance) in shaping the distribution of largely different (seed dispersers, pollinators and insectivorous) bird species that offer important ecological functions in these managed landscapes. For this purpose, we estimated bird densities at 22 forest patches during the spring of 2008 (see Fig. 1), and related them to the above descriptors of patch characteristics and configuration, obtained from a combination of field observations and telemetry analysis. Second, we use GIS-based models that incorporate the observed relationships between bird densities and spatial features of patches (i.e. a major component of habitat fragmentation *per se*), to estimate bird abundances at the landscape level for the present (2008) and a recent-past (1985) situations. Based on these two scenarios, we provide estimates of the relative impacts of forest loss and changes in spatial features of patches on local bird populations (i.e. on total bird abundances, aggregated over the whole landscape).

## Results

### Patch-level bird densities

GLM analyses detected significant relationships between bird density and patch features in four out of six species (Table 1), i.e. with the exception of thrush (no variables selected in the best model) and blackbird (best model included one significant variable, but the relationship was not robust, see below). For the other four species, patch isolation was the most common determinant of bird abundance (robust, significant or marginally-significant effects on three species; see below). Patch shape also had significant effects on three species (chucao, hummingbird and elaenia), while patch internal features (habitat characteristics) affected only one (chucao). Patch area did not affect clearly any of them (the only two significant effects were not robust; see below).

**Table 1:** Results of Generalized Linear Models for the effect of selected patch characteristics on patch-level bird density (parameter estimates ± standard error.

	DF	Area	P/A Ratio	Circle	Distance	Prox	DBH	Epiphytes	Flowers
Chucao†	9		0.0011±0.0004*	1.1676±0.4136 **	0.0008±0.0004*	0.0001±0.0001$	0.1923±0.0963**	−1.033±0.0118*	
Chucao‡	8		0.0009±0.0004*	1.1181±0.4189*	0.0008±0.0004*	0.00001±0.00001^NS^	0.1225±0.1313^NS^	−0.0291±0.0133**	
Elaenia	14		−1.493±0.0001*		0.0001±0.0001$				0.0001±0.0004^NS^
Hummingbird†	11	−0.0001±0.0001**		0.1608±0.0528**	−0.0001±0.00001**		−0.0204±0.0046**		
Hummingbird‡	10	0.0001±0.0001^NS^		0.1485±0.0616*	−0.0001±0.0001ns		−0.0181±0.005**		
Rayadito	14	−0.0001±0.0001^NS^			0.0005±0.0003$				0.839±0.2424^NS^
Blackbird†	9	−0.0001±0.0001**							−0.0012±0.002^NS^
Blackbird‡	8	0.0001±0.0002^NS^							−0.0007±0.00023^NS^

Only results from best-fitting models and species with at least one significant variable are shown. Note that we used an inverse link function; hence, positive parameters indicate negative effects and vice-versa.

**P<0.01,

**P<0.05,

$ P<0.10,

NS non-significant.

†Non-robust model (significant effects of certain variables were determined by single data points).

‡Robust model (1–2 data points excluded, see Methods for details).

**Figure 2:**
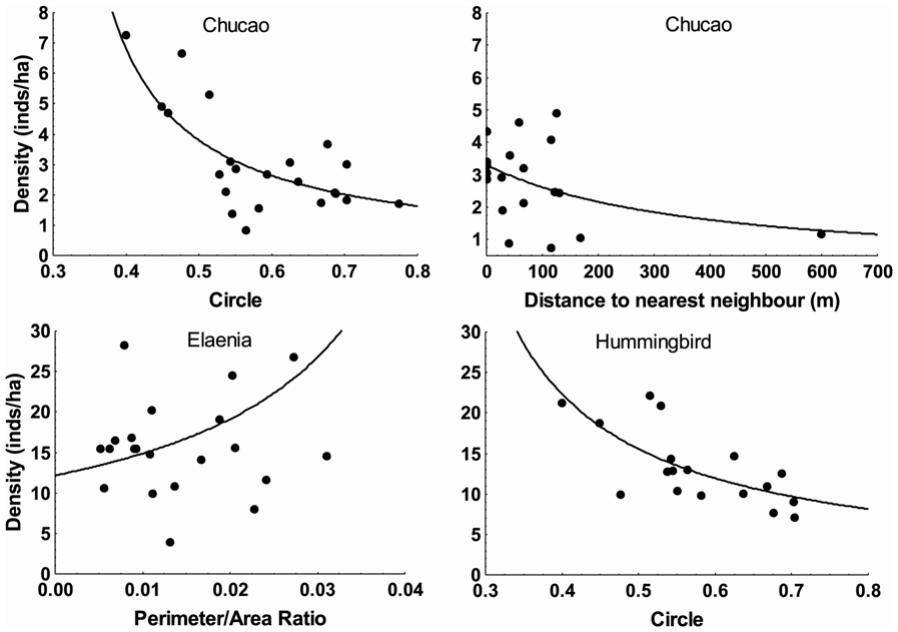
Partial residual plots showing the effects of patch characteristics on species densities (birds/ha). Effect of patch shape (CIRCLE and P/A Ratio) and connectivity (distance to the nearest neighbor) on chucao density (upper two panels), elaenia (lower left panel) and hummingbird (lower right panel). Filled circles represent values obtained after adding raw residuals to predicted values for each variable (assuming mean values for the remaining covariates), and then back-transforming the resultant value. Eliminating the apparent outlier for distance to the nearest neighbor did not change the model and hence it was retained (see Methods).

For chucao the best model included both measures of patch shape (P/A RATIO and CIRCLE), both measures of isolation (PROX and DIST) and two habitat variables (EPIPHYTES and DBH), but only four of these variables had significant effects (Table 1). Chucao densities declined with increasing patch elongation (CIRCLE) and isolation (DIST, Fig. 2). Although DBH showed a significant effect, partial residual plots indicated that the effect of tree size was largely caused by a single point (a DBH of 35 cm; Fig. S1) and disappeared when such point was removed from the dataset (p = 0.35); hence, we regard this relationship as unreliable and we will not discuss it further.

For elaenia the best model included a significant measure of patch shape (P/A RATIO), a marginally-significant measure of isolation (DIST,) and a non-significant habitat variable (FLOWERS; Table 1). Elaenia densities were higher in more irregularly-shaped patches (i.e. those with higher P/A ratios) and in less isolated ones (Fig. 2).

For hummingbird the best model fit included patch area (AREA), patch shape (CIRCLE), and isolation (DIST), as well as one habitat variable (DBH) (Table 1). However, inspection of the partial residual plots indicated that the relationships with area and isolation were largely determined by (different) single points located in the limit of the independent-variable measurement range (2465 ha for patch size and 599 m for DIST, Fig. S1). Models fit and selected after exclusion of each of these points did not include these variables; we will therefore discuss the results of the best model fit in which both points, hence both variables, are excluded. In such model, bird density increased with decreasing patch elongation (significant effect of CIRCLE; Fig. 2) and with increasing tree size (significant effect of DBH; data not shown).

For rayadito, we only found a marginally significant relationship between bird density and distance to nearest neighbor (DIST), in a best model that also included two non-significant variables, patch size (AREA) and number of flowers (FLOWERS) (Table 1). Rayadito density tended to decrease with increasing patch isolation.

The density of blackbird increased significantly with increasing patch area (AREA), in a best model that also included a non-significant variable, the abundance of epiphyte flowers (FLOWERS, Table 1). Partial residual plots indicated, however, that the effect of patch area was largely caused by a single data-point (the largest patch area measured: 2466 ha, Fig. S1), since it disappeared after excluding such data-point. We will therefore discuss only the latter model, which after selection only included the non-significant effect of FLOWERS.

### Point-level bird densities

GLMs carried out at the point level only showed significant relations between point-level variables and bird densities for two of the species sampled. Hummingbird density increased significantly from the patch edge towards its centre (Table 2, Fig. S2) and elaenia density increased significantly with epiphyte abundance (Table 2).

**Table 2:** Results of Generalized Linear Models for the effect of selected patch characteristics on point-level bird density (parameter estimates ± standard error).

	Circle	Distance	Distance to edge	Epiphytes	Flowers
Elaenia	4.21±23.17^NS^DF = 16			0.17±0.07***DF = 127	
Hummingbird	−8.33±15.64^NS^DF = 16		0.06±0.02***DF = 126		5.59±1.14***DF = 126

Only results from best-fitting models and species with at least one significant variable are shown.

**P<0.01,

*P<0.05,

$ P<0.10,

NS non-significant.

### Changes at landscape scale

Comparisons between variables extracted from the Landsat images showed that, from 1985 to 2008, the 8.82% reduction in forested area (from 55,837 ha to 50,914 ha) was accompanied by significant changes in all patch features (Table 3): patches became slightly smaller (16% decrease in patch size), much more isolated (58% increase in DIST and 73% decrease in PROX) and less irregular (50% and 11% decreases in CIRCLE and P/A RATIO).

**Table 3:** Summary statistics (average ± standard error) of the six variables used to quantify changes in patch features between 1985 and 2008.

	1985	2008	Mann-Whitney U
Patch area (ha)	94±42	79±20	114,862**
Patch shape (P/A Ratio)	0.02±0.0003	0.01±0.0002	67,268**
Patch shape (Circle)	0.63±0.004	0.56±0.004	95,821**
Distance to nearest neighbor (m)	47±2	74±3.6	144,124**
Proximity (100-m-buffer)	4040±584	1077±182	148,152**
Proximity (500-m-buffer)	5142±587	1304±186	151,640**
Number of fragments	574	588	

Patches smaller than 2.4 ha were not included in the analyses.

**P<0.01.

**Figure 3:**
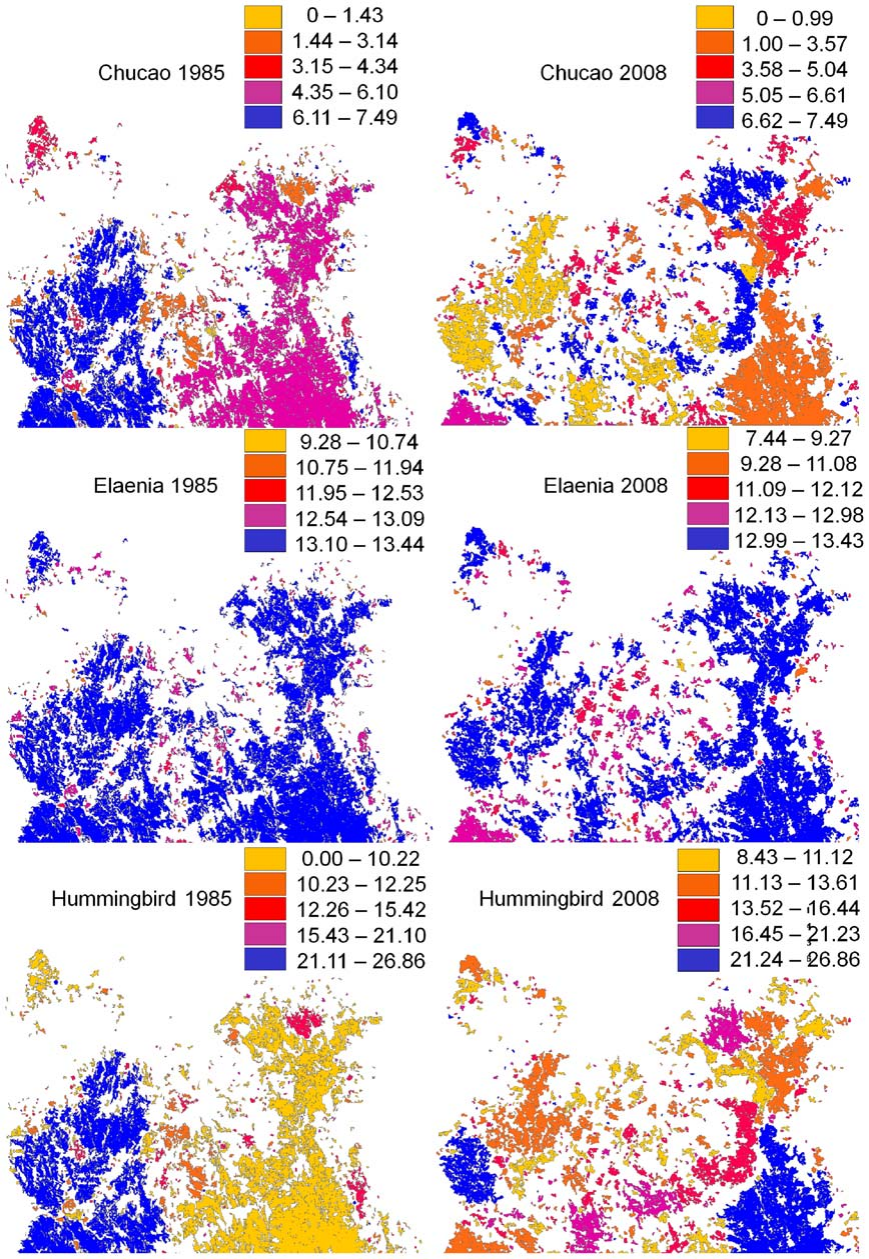
Estimated densities for three of the study species in 1985 and 2008. Calculations combine GLMs relating patch characteristics (size, shape and connectivity) to bird density, fitted to field data from 2008, and the analysis of patch characteristics based on satellite images from 1985 and 2008.

Landscape-level changes in bird densities (i.e. averaged across the whole study area) from 1985 to 2008 were estimated for the three species for which we obtained significant relationships between within-patch density and patch features (see above). It decreased significantly for one of the three species (white-crested elaenia), and increased for the other two (chucao and hummingbird) (Table 4, Fig. 3). The contrasting effects of patch-features changes on bird density mediated the overall effects of fragmentation on bird populations (i.e. landscape-level abundances) – since they exacerbated (elaenia), balanced (chucao) or overcompensated (hummingbird) the negative effects of forest loss (14% population decrease, for all bird species; Table 5). For chucao, the negative effect of forest loss was compensated by the positive effect of patch-feature changes (18% population increase), resulting in an almost null (<1%) decrease in total bird abundance. For elaenia, the negative effect of forest loss was exacerbated by the negative effect of patch-feature changes (3% population decrease), resulting in a stronger (17%) decrease in total bird abundance. For hummingbird, the negative effect of forest loss was compensated by a stronger effect of patch-feature changes (20% population increase), which resulted in a 3% increase in total bird abundance.

**Table 4:** Estimated density (individuals per hectare, average ± standard error) for three forest-bird species across all forest patches present in the study area (1214.75 km^2^, see Fig.1).

	1985	2008	Mann-Whitney U	Relative change
Chucao	4.11±0.86	4.84±0.11	90,266**	+17.8%
Elaenia	12.68±0.29	12.33±0.05	132,486**	**−2.8%**
Hummingbird	11.51±0.14	13.78±0.14	82,584**	+19.7%

**P<0.01.

## Discussion

Patch shape and isolation, rather than patch size, were the most important variables explaining the spatial (among-patches) variation in bird densities in fragmented, austral temperate forests. The internal features of the patches (i.e. local habitat) measured were of limited importance, affecting exclusively the density of hummingbird and chucao. Landscape-scale projections of these results for the three species significantly affected by the spatial configuration of patches (chucao, elaenia and hummingbird) indicated that the strong effects of forest loss on forest birds (estimated to result in 18 to 40% population decreases) can be exacerbated, balanced or compensated by the effect of patch-configuration changes. As a result, the effect of forest fragmentation on these bird populations ranged from negative to positive, depending on the relative contribution of the spatial configuration of patches (i.e. a major component of fragmentation *per se*) relative to forest loss (see table 5).

**Table 5:** Total bird abundance in the study area for six forest-bird species, due to (the various components of) forest fragmentation.

	Total bird abundance (^#^individuals)		Changes in bird abundance (% of initial abundance) caused by:			
	1985	2008	Forest loss (ΔF)	Changed patch features (ΔD)	Correlation term (ΔF*ΔD)	Forest Fragmentation (ΔF+ΔD+ΔF*ΔD)
Chucao	222,443	224,429	−31,864 (−14.3%)	+39,509 (+17.8%)	−5,659 (−2.6%)	+1,986 (+0.9%)
Elaenia	686,273	571,738	−98,305 (−14.3%)	−18,943 (−2.8%)	+2,713 (+0.4%)	−114,535 (−16.7%)
Hummingbird	622,950	638,974	−89,234 (−14.3%)	+122,858 (+19.7%)	−17,599 (−2.8%)	+16,025 (+2.6%)
Rayadito	935,236	801,268	−133,968 (−14.3%)	-	-	−133,968 (−14.3%)
Blackbird	342,595	293,520	−49,075 (−14.3%)	-	-	−49,075 (−14.3%)
Thrush	1,381,746	1,183,817	−197,928 (−14.3%)	-	-	−197,928 (−14.3%)

All estimates are based on relationships between bird density and patch features measured in the 2008 field survey, and patch features measured in Landsat images from 1985 and 2008. For the three species in which bird density did not vary with patch characteristics, changes in bird abundance caused by changed patch features (ΔD) and the correlation term (ΔF*ΔD) are predicted to be zero, thus they are left blank. Sign before the figures indicates decreased (−) or increased (+) bird abundances.

Our results contrast with previous studies that reported a predominant effect of the size of remnant patches over bird occurrences (e.g. [11], [13], [24]; but see [9] for a general review of patch-area effects and [12] for meadow birds) and suggest that (other patch features being equal) large isolated patches may maintain bird populations of the species surveyed comparable to those in an equivalent area of small connected patches. This divergence may arise from three differences in the study systems and/or in the methodology used in the bird surveys. First, we have measured a smaller number of bird species (six) than other studies (55 species in [25]), although other studies focused on less (one or two species) have predominantly reported significant effects of patch size [26]. The medium to small size of our study species might also have resulted in a downward bias on the effect of patch area, although previous results with similar-sized species suggest this is not the case ([17] and references therein). Second, previous studies measured the persistence of species in focal areas (presence-absence of single species or species richness per site) rather than differences in species densities or population sizes (as reported here). It is however worth noting that, even if bird density does not change with patch area, it is reasonable to expect a decrease in persistence in small patches, because local population size would decrease in direct proportion to patch area - thus small-patch populations will be more prone to stochastic extinctions and/or Allee effects. Third, we chose to survey fragments larger than 2.1 ha, estimated to be large enough to support a single pair of individuals of our study species; while the negative effects of patch area reduction reported by Willson et al. [24], who compared very large (100–350 ha) with tiny patches (<0.5 ha), probably reflect the loss of species in patch sizes too small to cover their minimum requirements (e.g. minimum territory size is estimated to be 0.8 to 1.3 ha for *Scelorchilus rubecula* in fragmented areas, [27], [28]). At any rate, even if we failed to detect effects at very small patches (<2.1 ha), our data show that over a broad range of variation (from 2.1 to 2,465 ha, thus spanning a difference of 3 orders of magnitude, see Table S1), patch size did not affect significantly the density of our focal bird species. This difference is smaller (from 2.1 to 433 ha) for the two species (blackbird and hummingbird) for which we eliminated the larger patch (since its inclusion resulted in non-robust relationships). It is therefore possible that there are effects, undetected here, above such 433 ha limit – though such limit is well above the range of patch sizes addressed by most published studies, and leaves out most remnant patches found in our study area.

Instead, we found patch shape and isolation to be the most important predictors of bird density in forest fragments. The effect of patch shape and isolation varied, however, among species. Patches with longer edges relative to their area (i.e. elongated and irregular shapes) showed higher densities of elaenias, but lower densities of chucaos and hummingbirds; while isolated patches showed lower densities of chucaos and elaenias. This variation may reflect differences in the specieś ecology, particularly concerning their ability to utilize patch edges and the intervening habitat matrix - and therefore move between distant patches. Hummingbirds, for example, feed preferentially inside the forest fragments (as indicated by their lower abundance in or near fragment edges; Fig. S2), probably as a consequence of the higher abundance of *Mitraria coccinea* far from the forest edge (Magrach et al,. unpublished data), which is their main floral resource at this time of the year. However, they are also able to utilize the abundant flower resources available in the bushes of the intervening matrix (Magrach, unpubl.data); hence, they were not affected by patch isolation (Table 1). Chucao and rayadito, respectively a strict forest-dwelling and a poor-dispersing species ([29], [30], [31]), were negatively affected by irregularly-shaped patches (i.e. with abundant forest edges) and patch isolation. White-crested elaenia, a migrant species with greater dispersal capabilities [32], showed increased densities in patches with longer edges.

Our comparisons between forested areas at two different times (1985 and 2008) showed differences in all variables measured. We observed a decline in mean patch size as well as an increase in the number of patches, two of the main components of the fragmentation process [33]. Patch shapes also became more regular and compact, probably due to higher logging at forest edges, and more isolated, with larger distances to the nearest patch and less or smaller neighboring patches.

During the same period (from 1985 to 2008), estimated bird densities decreased significantly for one of the species (elaenia) and increased for another two (chucao and hummingbird). These effects are independent of total area loss, since they refer to bird densities (number of individuals per unit area), not to their abundances. They reflect the interplay between the specific changes in patch features and the specific requirements of the different species. Elaenias show higher densities at irregularly-shaped and well-connected patches; they are disfavored by the trend towards more regular, compact and isolated patches that characterized the period 1985–2008, although the net effect is fairly small (a 3% decrease). Chucao density is strongly favored by the trend towards more regular and compact patches; this positive effect dominates over the negative effect of connectivity loss, yielding an 18% bird-density increase as a result. This apparent increase can be interpreted as a “refugee effect” (sensu [34]), with an increased number of individuals becoming ‘cornered’ in increasingly smaller and more isolated patches that are losing their irregular periphery due to logging from the edges. In contrast with these two species, hummingbirds seem to be slightly favored by the fragmentation process, owing to their insensitivity to patch isolation and their preference for more regular and compact patches. The thriving capabilities of hummingbirds to persist in managed landscapes is not new to ecological studies ([35]–[37] and references therein); for example, several authors have reported that the increased numbers of flowers (therefore nectar resources) in forest clearings may confer them advantages over other species ([38]–[39]).

Forest loss was not always the main driver of bird population changes (i.e. bird abundances at landscape scale). It was indeed the only factor (within the scope of this study) driving population changes for the three species unaffected by changing patch features (rayadito, blackbird and thrush), which therefore showed population declines of 14% (i.e. directly proportional to habitat loss). But for the three species significantly affected by changes in patch features, forest loss only showed dominant effects in one case (14% out of a 17% decrease in elaenia); for the other two, the effects of changes in patch features equaled (chucao) or surpassed (hummingbird) the effects of forest loss. As a result, the overall effect of fragmentation was negative for one of the species (elaenia), neutral for another (chucao, though it probably reflects a refugee effect), and positive for another (hummingbird). These results suggest that, as forest loss and fragmentation increase, all species but those that combine a high dispersal capability and enough plasticity to use the intervening matrix (in our case, the hummingbird) will face population losses. The predictions of forest loss available for our study area (3% for the period 2010–2020) [40] also suggest that such population losses will be accentuated considerably in the near future.

Our study shows that, in temperate austral forests, fragmentation processes have local and landscape-level effects on bird densities and abundances that modulate the direct effect of (total) forest loss. These effects are mediated by changes in patch shape and connectivity, rather than by patch size. Hence, in addition to trying to reduce forest loss to a minimum, forest-management and nature-conservation plans should not overlook the possible tampering effect of an adequate spatial management of logged areas. With the aim of optimizing the connectivity of logged forests, spatially-explicit models forecasting the response of different target species to patch abundance, shape and configuration could be used to inform and guide planning and decision-making.

## Materials and Methods

### Study system

The study was carried out at Isla Grande de Chiloé, southern Chile (42°00′S, 73°35′W, Fig. 1), within a mosaic of old-growth and second-growth forest patches embedded in a matrix of pastures and crops. Original vegetation is a mixture of Valdivian and North Patagonian temperate rain forests (a globally endangered ecoregion; [40]) dominated by broad-leaved evergreen species with an abundant understory of vines, epiphytes and dense bamboo thickets (*Chusquea* sp.), numerous logs and snags, and shrub-dominated degradation stages in gaps and open areas (see [41]). Annual precipitation is 2,124 mm (for the period 1996–2008) with temperatures ranging from 2.5°C to 17.5°C (monthly averages). The area is characterized by a highly endemic flora and fauna, with e.g. 13 endemisms out of 44 native bird species [42].

Temperate forests in southern Chile have been subjected to great anthropic pressure, with forested area being reduced to 41% of its initial cover in 1999 and an additional reduction to 35% predicted for 2020 [40]. Deforestation is mainly due to the expansion of crops and pasturelands [43].

Our study focused on six of the most abundant and singular species of Chilean temperate forests (more than 80% of the avifauna of Chiloean forests was composed of five of these species [44], and three of them are endemic to this area 24): chucao tapaculo (*Scelorchilus rubecula*), white-crested elaenia (*Elaenia albiceps*), green-backed firecrown hummingbird (*Sephanoides sephaniodes*), thorn-tailed rayadito (*Aphrastura spinicauda*), austral blackbird (*Curaeus curaeus*) and austral thrush (*Turdus falcklandii*). They provide important ecological functions, including seed dispersal (four species) and pollination (two species, one of which is also a seed disperser, *Elaenia albiceps*), and also include a strictly insectivorous species. The chucao is an understory resident bird that typically consumes both insects and fruits [44]. It is one of the 13 bird species endemic to the south-temperate forests [42] and has been described as a species with limited dispersal constrained by open habitat [30]. The elaenia is a migrant species present in the study area from October to March. It is one of the main seed dispersers in the study area [46], although it can also feed on nectar and insects during part of its life cycle, acting as an important pollinator for some species [47]. It is able to use the surrounding bush matrix to some extent, although its abundance is greatly reduced in matrix areas relative to forest (0.10 vs. 0.98 individuals per sampling station, [44]). The hummingbird is the only hummingbird species found in Chiloé Island and the main pollinator for many plant species, amongst others the majority of vines and epiphytes in the study area [48]. It is the most abundant bird in the area (representing up to 23% of the forest birds, [44]) and, though it uses forests preferentially and depends on them for refuge and breeding [49], it can be locally abundant in the surrounding matrix [44]. The rayadito is a resident insectivorous species endemic to the temperate forests of South America, where it has been classified as a large-tree user [29]. Recent studies have shown that it has reduced dispersal capabilities when remnant patches are not connected by forest [50]. The blackbird is a common species in Chilean temperate forests, and shows a mixed diet of fruits and invertebrates [51]. Its abundance has been observed to increase near forest edges but not in the intervening matrix (0.16 vs. 0.40 individuals per sampling station in forest interiors vs. forest edges, respectively; [44]). The thrush represents, together with the elaenia, the main resident avian frugivore in this type of forests [45]. This species has great dispersal capabilities that allow it to have comparable abundances in forests and bush areas (0.94 vs. 0.90 individuals per sampling station, respectively; [44]).

### Bird surveys

Bird surveys were conducted in 22 forest fragments chosen to differ in size, shape and degree of isolation (see Table S1). We selected a minimum patch size of 2 ha, aimed at excluding patches too small to accommodate the home range of a single pair of the focal species, based on published data on home-range size of the Chucao (0.8–1.3 ha; [24], [28]) and the Huet-Huet (*Pteroptochos castaneus*, a species slightly smaller than the blackbird and the thrush; 3.08 ha, [28]). However, owing to various practicalities of the field work (e.g. patch accessibility and permits by land owners), the smallest patch sampled finally had 2.4 ha. Surveys were conducted from mid-October to mid-December using variable-distance cue-count point surveys [52]. We selected this technique because it provides unbiased density estimates when low numbers of species are surveyed [53], as is the case here, and it is suitable for closed habitats with high canopies, like our study area. To prevent confounding effects on the number of individuals detected, surveys were always carried out by the same observer (A.M.), under comparable weather conditions (i.e. avoiding rainy or windy days) and at the same time of the day (from 06:00 to 13:00 h, similar to [24], [48]).

At each forest patch, we located 8 census points: four (hereafter referred to as “patch-centre”) points were situated at 100 m from the nearest edge and separated by a minimum distance of 100 m from each other; the other 4 points were located between each of the previous points and the forest edge, and randomly assigned to four distances to such edge (one each): 0, 25, 50 and 75 m.

Each census point was surveyed once. We allowed 5 minutes for birds to settle between our arrival and the start of the bird count, and then recorded for 8 minutes every cue (visual or songburst) belonging to the surveyed species, together with an estimate of the distance between the registered cue and the observeŕs point [52]. (To ensure the reliability of distance estimates, the observer performing the survey undertook a period of training in one of the fragments surveyed, using a laser-based rangefinder (Nikon 550 AS) to compare the estimated distances with reference measures). To account for possible differences in the detection probability, we also measured the number of trees present in a 10-m-strip around each census point and introduced it as a covariable in the analyses (see below).

To obtain reference values of cue-rates for each species surveyed, we also measured the number of songbursts produced per time unit in a subsample of focal individuals whose position allowed for clear, simultaneous visual observations during at least 5 minutes.

### Epiphyte surveys

Surveys were carried out for all angiosperm epiphytes encountered in the study area (*Luzuriaga polyphilla*, *Luzuriaga radicans*, *Mitraria coccinea*, *Sarmienta repens*, *Asteranthera ovata*, *Campsidium valdivianum*, *Tristerix corymbosus* and *Fascicularia bicolor*). We included these species in the characterization of forest patches because they account for 27% of all plants bearing fleshy fruits [55] and represent 67% of the flowers visited by the hummingbird *S. sephaniodes* [48] in Chiloé Island.

At each patch, we recorded every epiphytic plant located within 100 m (1 m-wide) transects connecting the four patch-centre, bird-census points with the nearest patch edge (i.e. four transects per patch). For each tree included in the transect, we recorded the diameter at breast height (DBH) and registered the species identity and abundance of its epiphytes, measured (as required by the epiphytés architecture) as the number of individuals or ramets per host tree. Trees with DBH<5 cm were not considered. For flowering or fruiting epiphytes, we also recorded the number of flowers or fruits per individual. Epiphytes located in high areas of the trees were surveyed with the aid of binoculars (Nikon 8*40). To limit the number of variables introduced in the models and avoid problems of multi-colinearity, we used aggregated measures of epiphyte abundance and flower and fruit production (i.e. all species pooled).

### Landscape and patch metrics

Patch metrics were obtained from two Landsat TM images, one considered to be contemporaneous to the bird surveys (February 18, 2008), and another one representing a record 23 years older (January 25 1985). Images were analyzed using an isodata, non-supervised algorithm to discriminate forested and non-forested areas (based on a 20-class categorization; Idrisi 15.0, Andes Edition, Clarks Lab). The resulting layer of forested areas was then analyzed using FragStatsBatch for Arcgis 9 [54], [55] and V-LATE 1.1 for Arcgis 9 [56] to produce a series of descriptive measures for each of the 22 forest fragments included in the bird surveys. The following measures were used: patch size, two measures of patch shape (CIRCLE and P/A RATIO) and two measures of patch isolation (DISTANCE and PROX). CIRCLE was defined as 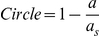 where *a* is patch area and *a_s_* is the area of the smallest circle circumscribing the patch. It takes values of 0 for circular patches and tends to 1 for elongated, linear patches one-cell wide [54]. P/A RATIO is the ratio between patch perimeter and area and increases as patch shape becomes more irregular. DISTANCE is the Euclidean distance to the nearest neighbor. PROX is defined as 
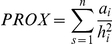
 where *a_i_* is the area of every patch falling within specified neighborhood “buffers” of the focal patch (see below) and *h^2^_i_* is the edge-to-edge distance between each patch *i* and the focal patch [54]. After preliminary calculations for different buffer sizes around the focal patches (ranging from 100 to 2000 m), we selected the buffer size at which the value of the proximity index saturated (500 m) and a smaller value assumed to represent the patch-crossing distance of a poor-flying forest-bird species (100 m, similar to the distance used by [28] for the chucao).

### Statistical analyses

We estimated bird densities using the MCDS module from program Distance 5.0 ([57], one project per species). For each bird species, we fitted a global model with patches as strata and the mean number of trees per transect as a covariable. We also included species cue-rate to obtain bird densities from the number of cues. We repeated the same procedure for point-level estimates of bird densities, this time using the number of trees in the 10 m transect-segment around each surveyed point as the covariable. The estimation of point-level densities involves a previous estimation of a patch-level detection function, from which a density value per point is estimated [57].

We obtained global model fits for the two key functions available in MCDS engine (half-normal and hazard-rate with different adjustment terms), selected the best-performing model based on their AIC value and used such model to carry out bootstrap calculations of global-level variance (based on 1000 resamples) and to estimate patch-level bird densities [58].

The effect of forest patch characteristics over bird–density estimates at the patch level was analyzed by means of generalized linear models, using the Genmod procedure available in SAS 9.1 (SAS, SAS Institute, Gary, NC, 2002–2003). We fitted one separate model for each bird species using the gamma error distribution and the inverse link function. The analyses were performed at both patch and point level. At patch level, initial models included patch area, patch shape (two variables: CIRCLE and P/A RATIO) and isolation (two variables: DISTANCE and PROX), and three descriptors of within-patch habitat features (mean tree DBH; mean abundance of epiphyte plants per tree; and mean abundance of epiphyte flowers or fruits per tree, depending on the bird's diet). No interaction was included in the models. All these variables showed low levels of colinearity (r<0.65 for all pair-wise correlations; Table S2), except for the two proximity measures. Model reduction was then achieved by fitting the complete family of models (full-model, excluding interactions between variables, plus all the potential subsets of independent variables) and selecting the best-performing one on the basis of their respective AICc scores. Whenever any two variables were significantly correlated, they were not included in the same model; instead, we used separate models including one or the other variable, and selected the best one using their AIC score.

Bird-density estimates at point-level were fitted to generalized linear mixed models, using the Glimmix procedure also available in SAS 9.1 (SAS, SAS Institute, Gary, NC, 2002–2003). The models included four patch-level variables (AREA, CIRCLE, P/A RATIO, DISTANCE and PROX), three habitat features measured at each surveyed point (distance to the nearest edge; mean tree DBH; mean abundance of epiphyte plants per tree; and mean abundance of epiphyte flowers or fruits per tree, depending on the bird's diet) and the random factor “patch”. For every model we fit a normal distribution with an identity link function. No interactions were included in the models. Detection of correlated variables and model selection were done as before.

Whenever the graphical exploration of the model suggested that an effect could be caused solely by one or two outliers, we repeated the analyses eliminating these outliers; if the effect disappeared, we retained the model without the outliers. To obtain estimates of total bird abundance at the landscape level, we used the landscape and patch-level metrics obtained from the 2008 and 1985 Landsat TM images of our study area (which included a surface of 1214.75 km^2^). To be able to interpolate the results of the surveys (see below), we restricted our analyses to forest patches falling within the range of areas, shapes and degrees of isolation observed there (e.g. we discarded patches under 2.4 ha). Based on the parameters obtained in the GLMs, we estimated the expected density of each bird species for each patch identified in the Landsat images (only for the three species for which we found significant effects of the variables measured in forest fragments). Average density estimates of each species for the two years of study were compared by means of Mann-Whitneýs U, using the software SPSS 16.0.

Total bird abundances for 1985 and 2008 (A_1985_ and A_2008_, respectively) were then calculated for the six species sampled by multiplying average bird densities (D_1985_ and D_2008_) by total forest covers (i.e. the cumulative surface of all forest patches larger than 2.4 ha, F_1985_ and F_2008_) at their respective years. The difference provided estimates of changes in total bird abundances caused by forest fragmentation at landscape level, which were further decomposed into the relative contributions of forest loss and (changes in) patch features, as follows:



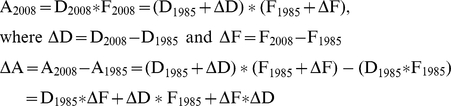
Hence, changes in bird abundance result from the additive effects of: (1) changes in total forest cover (D_1985_*ΔF), i.e. forest loss; (2) changes in bird density (ΔD*F_1985_) resulting (in our specific calculations) from changes in patch features; (3) an interaction term (ΔF*ΔD) that reflects the correlation between changes in forest cover and changes in bird densities (i.e. patch features). It is important to note that this scenario-building exercise assumes (as commonly done when projecting scenarios into both past and future; e.g. [25], [59]–[61]) that bird densities responded similarly to patch features in 2008 and in 1985 (i.e. that we could predict bird responses to patch features in 1985 using data measured in 2008).

## Supporting Information

Figure S1 Partial residual plots showing the effects of area, connectivity (distance to the nearest neighbor) and mean tree DBH over (birds/ha) hummingbird (upper two panels), chucao (lower left panel) and blackbird (lower right panel) densities (birds/ha). Filled circles represent values obtained after adding raw residuals to predicted values for each variable (assuming mean values for the remaining covariates), and then back-transforming the resultant value. Triangle shows outlier values that render the relationship non-robust.
(TIF)

Figure S2 Partial residual plot showing the effect of the distance to the nearest edge on point-level density of hummingbird (birds/ha). Filled circles as in Figure S1.
(TIF)

Table S1 Values for the variables measured at the patch level for each of the 22 patches sampled.
(DOCX)

Table S2 Bivariate correlations for the variables measured. **P<0.01, *P<0.05.
(DOCX)
