# Supplementary figures and images for: Changes in Patch Features May Exacerbate or Compensate for the Effect of Habitat Loss on Forest Bird Populations
Source: PLoS One. 2011 Jun 28;6(6):e21596. doi: 10.1371/journal.pone.0021596 (PMC3125244; doi:10.1371/journal.pone.0021596)

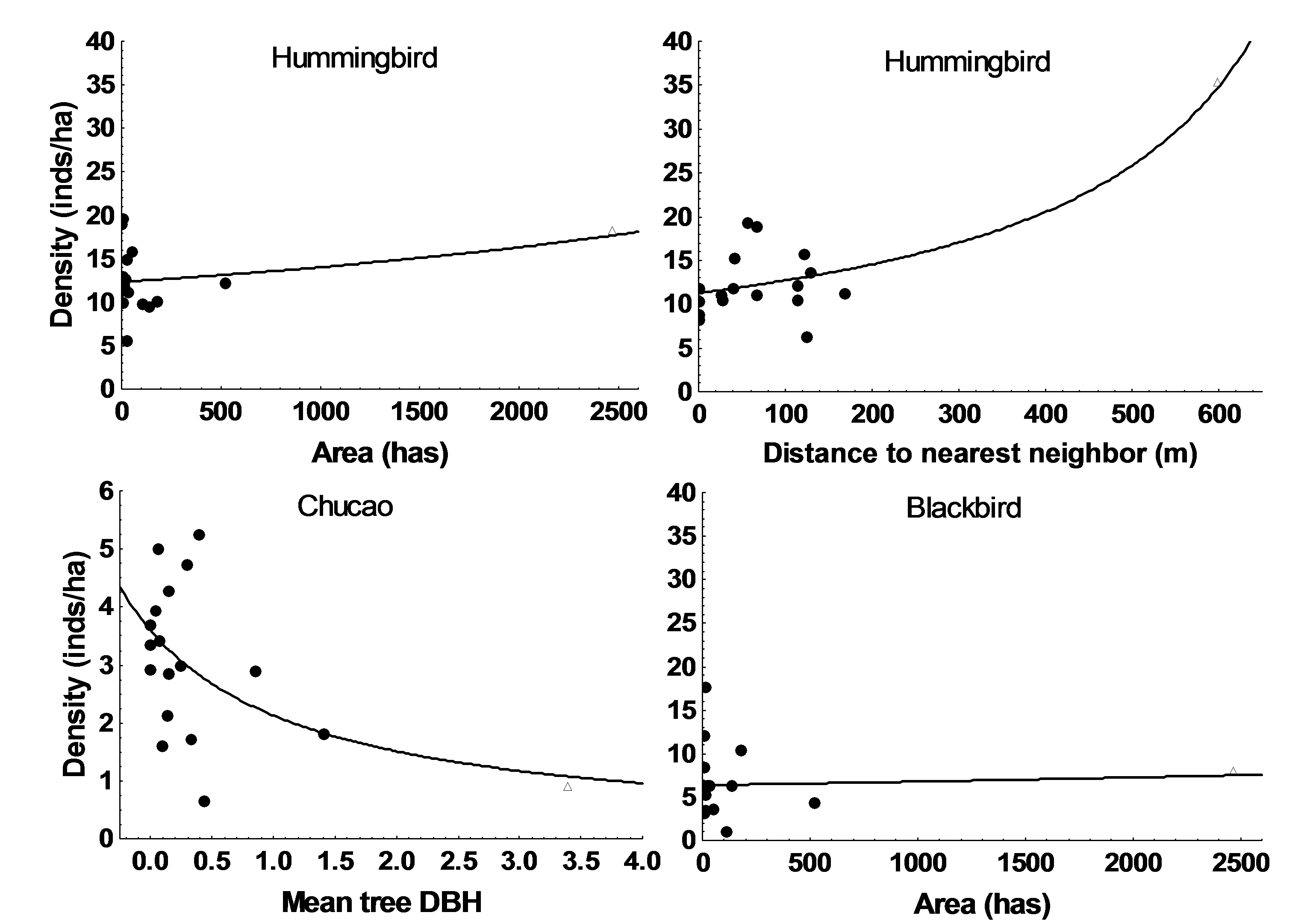

Supplement: Figure S1 — Partial residual plots showing the effects of area, connectivity (distance to the nearest neighbor) and mean tree DBH over (birds/ha) hummingbird (upper two panels), chucao (lower left panel) and blackbird (lower right panel) densities (birds/ha). Filled circles represent values obtained after adding raw residuals to predicted values for each variable (assuming mean values for the remaining covariates), and then back-transforming the resultant value. Triangle shows outlier values that render the relationship non-robust. (TIF) [file pone.0021596.s001.tif]

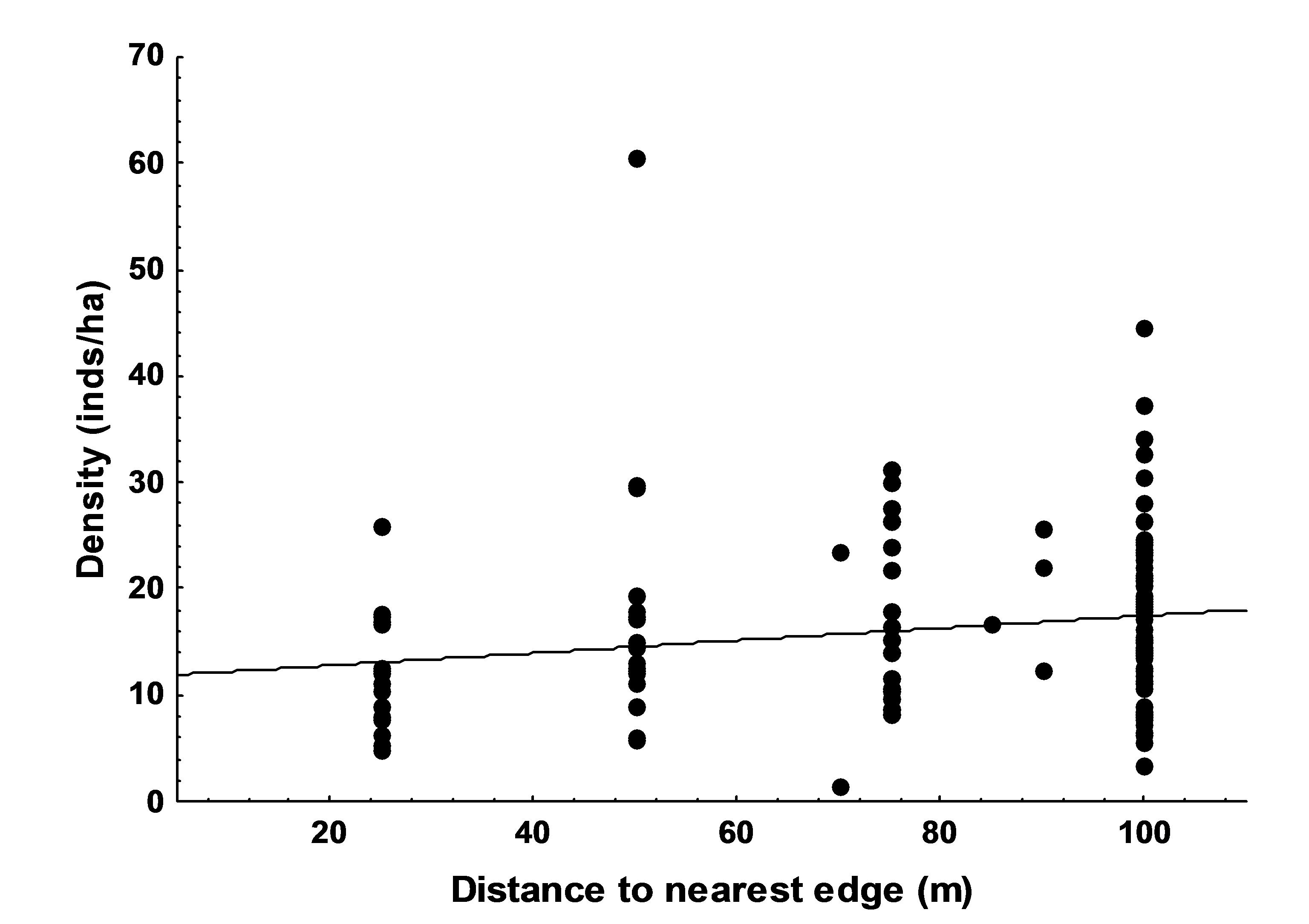

Supplement: Figure S2 — Partial residual plot showing the effect of the distance to the nearest edge on point-level density of hummingbird (birds/ha). Filled circles as in Figure S1. (TIF) [file pone.0021596.s002.tif]
