# Supplementary material for: Changes in Patch Features May Exacerbate or Compensate for the Effect of Habitat Loss on Forest Bird Populations
Source: PLoS One. 2011 Jun 28;6(6):e21596. doi: 10.1371/journal.pone.0021596 (PMC3125244; doi:10.1371/journal.pone.0021596)
Supplement: Table S1 — Values for the variables measured at the patch level for each of the 22 patches sampled. (DOCX) [file pone.0021596.s003.docx]

**Table S1.** Values for the variables measured at the patch level for each of the 22 patches sampled.

| Fragment | Area (ha) | P/A Ratio | Circle | Distance to the nearest neighbor (m) | Proximity (100-m-buffer) | Proximity (500-m-buffer) |
| --- | --- | --- | --- | --- | --- | --- |
| Anibal | 2.03 | 0.036 | 0.40 | 66.3 | 7.95 | 24.42 |
| Arizmendi | 18.36 | 0.014 | 0.45 | 129.39 | 0.00 | 1525.02 |
| Canelo | 136.78 | 0.009 | 0.55 | 0 | 0.00 | 1253.61 |
| Caulin | 885.68 | 0.006 | 0.59 | 0 | 74.89 | 266.64 |
| Duran | 519.68 | 0.007 | 0.64 | 0 | 28543.12 | 28581.97 |
| Gallardo | 51.17 | 0.011 | 0.62 | 40.98 | 1091.94 | 1103.67 |
| Hella | 10.80 | 0.021 | 0.70 | 26.13 | 3018.84 | 3021.35 |
| Juvenal | 3.49 | 0.031 | 0.53 | 65.4 | 4.85 | 14.47 |
| Koch | 2465.99 | 0.006 | 0.67 | 0 | 6061.14 | 6216.84 |
| Konpatski | 5.20 | 0.024 | 0.54 | 599.24 | 0.00 | 0.00 |
| Konpatski II | 30.54 | 0.011 | 0.54 | 28.09 | 13.16 | 17.71 |
| Linebrick | 14.05 | 0.017 | 0.56 | 40.05 | 156.92 | 165.10 |
| Llanquihue | 134.67 | 0.009 | 0.69 | 0 | 11.82 | 16.27 |
| Minert | 25.59 | 0.013 | 0.48 | 124.08 | 0.00 | 43.75 |
| Ojeda | 9.58 | 0.020 | 0.68 | 114.34 | 0.00 | 19.92 |
| Ojeda U | 3.98 | 0.027 | 0.55 | 114.34 | 0.00 | 10.50 |
| Ostracodo | 108.84 | 0.008 | 0.58 | 167.74 | 0.00 | 464.00 |
| Quilar | 257.65 | 0.005 | 0.46 | 0 | 190.93 | 198.18 |
| Rapaport | 175.61 | 0.011 | 0.70 | 0 | 27.01 | 148.52 |
| Senda | 433.17 | 0.009 | 0.77 | 0 | 885.92 | 924.98 |
| Ulloa-Delgado | 24.37 | 0.019 | 0.69 | 121.94 | 0.00 | 427.09 |
| Zuñiga | 5.52 | 0.023 | 0.51 | 56.77 | 13.50 | 1501.20 |
