# Supplementary material for: Changes in Patch Features May Exacerbate or Compensate for the Effect of Habitat Loss on Forest Bird Populations
Source: PLoS One. 2011 Jun 28;6(6):e21596. doi: 10.1371/journal.pone.0021596 (PMC3125244; doi:10.1371/journal.pone.0021596)
Supplement: Table S2 — Bivariate correlations for the variables measured. **P<0.01, *P<0.05. (DOCX) [file pone.0021596.s004.docx]

**Table S2.** Bivariate correlations for the variables measured. ** P<0.01, * P<0.05.

|  | Patch area (ha) | Patch shape (P/A Ratio) | Patch shape (Circle) | Distance to nearest neighbor (m) | Proximity (100-m-buffer) | Proximity (500-m-buffer) | Epiphyte abundance | Number of flowers | Tree DBH |
| --- | --- | --- | --- | --- | --- | --- | --- | --- | --- |
| Patch area (ha) |  | **-0.45*** | 0.28^NS^ | -0.26^NS^ | 0.30^NS^ | 0.30^NS^ | 0.15^NS^ | 0.18^NS^ | -0.13^NS^ |
| Patch shape (P/A Ratio) |  |  | -0.35^NS^ | -0.37^NS^ | -0.26^NS^ | -0.27^NS^ | 0.13^NS^ | 0.07^NS^ | -0.32^NS^ |
| Patch shape (Circle) |  |  |  | -0.22^NS^ | 0.20^NS^ | 0.17^NS^ | -0.31^NS^ | 0.03^NS^ | 0.20^NS^ |
| Distance to nearest neighbor (m) |  |  |  |  | -0.18^NS^ | -0.18^NS^ | -0.06^NS^ | -0.09^NS^ | -0.17^NS^ |
| Proximity (100-m-buffer) |  |  |  |  |  | **0.99***** | 0.14^NS^ | -0.13^NS^ | 0.32^NS^ |
| Proximity (500-m-buffer) |  |  |  |  |  |  | 0.13^NS^ | 0.35^NS^ | -0.17^NS^ |
| Epiphyte abundance |  |  |  |  |  |  |  | **-0.54^*^** | -0.42^NS^ |
| Number of flowers |  |  |  |  |  |  |  |  | -0.04^NS^ |
| Tree DBH |  |  |  |  |  |  |  |  |  |
